# DnaJ/Hsp40 Family and Parkinson's Disease

**DOI:** 10.3389/fnins.2017.00743

**Published:** 2018-01-10

**Authors:** Takafumi Hasegawa, Shun Yoshida, Naoto Sugeno, Junpei Kobayashi, Masashi Aoki

**Affiliations:** Division of Neurology, Department of Neuroscience and Sensory Organs, Tohoku University Graduate School of Medicine, Sendai, Japan

**Keywords:** DnaJ protein, Hsp40, co-chaperones, Parkinson's disease, neurodegeneration

## Abstract

Parkinson's disease (PD) is the second most common devastating neurodegenerative disorder after Alzheimer's disease. The precise molecular and cellular basis underlying PD still remains uncertain; however, accumulating evidence suggests that neuronal cell death is caused by a combination of environmental and genetic factors. Over the previous two decades, more than 20 genes have been identified as the cause of and/or risk for PD. Because sporadic and familial forms of PD have many similarities in clinical and neuropathological features, common molecular pathways, such as aberrant mitochondrial and protein homeostasis, are likely to exist in both conditions. Of the various genes and proteins involved in PD, the versatile DnaJ/Hsp40 co-chaperones have attracted particular attention since several genes encoding this protein family have been successively identified as the cause of the familial forms of PD/Parkinsonism. In this review, we will introduce the current knowledge regarding the integratory and modulatory effect of DnaJ/Hsp40 in various cellular functions and argue how the failure of these proteins may initiate and/or facilitate of the disease.

## Introduction

Newly synthesized proteins are transported to the destination site where they exert distinct functions; however, some proteins fail to properly fold, and misfolded proteins tend to form aggregates that may be potentially harmful to cells. To combat these perpetual threats, cells have evolved dexterous quality control mechanisms that facilitate degradation by the ubiquitin-proteasome system and autophagy-lysosome pathway or refolding of misfolded proteins to normal tertiary structures by molecular chaperones (Oshima et al., 2016; Ciechanover and Kwon, 2017). The most well-known molecular chaperones are heat shock proteins (HSPs), which were named after the phenomenon of puffing in larval salivary gland chromosomes of *Drosophila* following heat exposure (Pauli et al., 1992). Heat shock and numerous noxious stimuli, including UV, oxidative stress, hypoxia, osmotic stress, and heavy metals, induce the expression of HSPs, which is considered as a fundamental biological defense mechanism (Piano et al., 2004). The family of HSPs are evolutionarily conserved proteins across species and are classified based on their molecular weights; different HSPs have distinct yet partially overlapping functions. For example, Hsp90, which is known to be one of the most abundantly expressed proteins (1–2% of total soluble proteins in an unstressed condition) in the cytosol, forms a complex with its client proteins, such as cell surface receptors, transcription factors, and protein kinases, to modulate their functions (Schopf et al., 2017). Thus, Hsp90 plays pivotal roles in various cellular processes, such as proliferation, differentiation, carcinogenesis, and neurodegeneration. Several Hsp90 inhibitors, including the geldanamycin and its analogs demonstrate anti-tumor effects in various cellular and animal models (Soga et al., 2013). The Hsp70 family is the eukaryotic homolog of the bacterial molecular chaperone DnaK and is widely expressed in various tissues and organs. The members of the Hsp70 family are characterized by their expression levels, activities, and subcellular distribution (Zuiderweg et al., 2017). Under physiological conditions, Hsp70 mainly resides in the cytoplasmic space, whereas in response to stress, it is upregulated and relocates from the cytosol to the nucleus and nucleoli. The heat shock cognate 70 (Hsc70) is, on the other hand, defined by its constitutive expression and cytoplasmic localization. Another Hsp70 member, BiP (also referred to as a 78-kDa of glucose-regulated protein: GRP78), expresses in the endoplasmic reticulum (ER) and acts as a chaperone for secreted or membrane proteins (Dudek et al., 2009). Hsp70 binds and shields the hydrophobic peptides of client proteins in an ATP-dependent manner, thus preventing protein aggregation and proper folding (Zuiderweg et al., 2017). Hsc70 chaperone mediates the lysosomal targeting of substrates with a KFERQ-like sequence (i.e., chaperone-mediated autophagy; Cai et al., 2015). Furthermore, Hsp70 is involved in the intracellular traffic of membranes, including the involvement in regulation of endocytosis and exocytosis mechanisms (Goldfarb et al., 2006).

**Figure 1:**
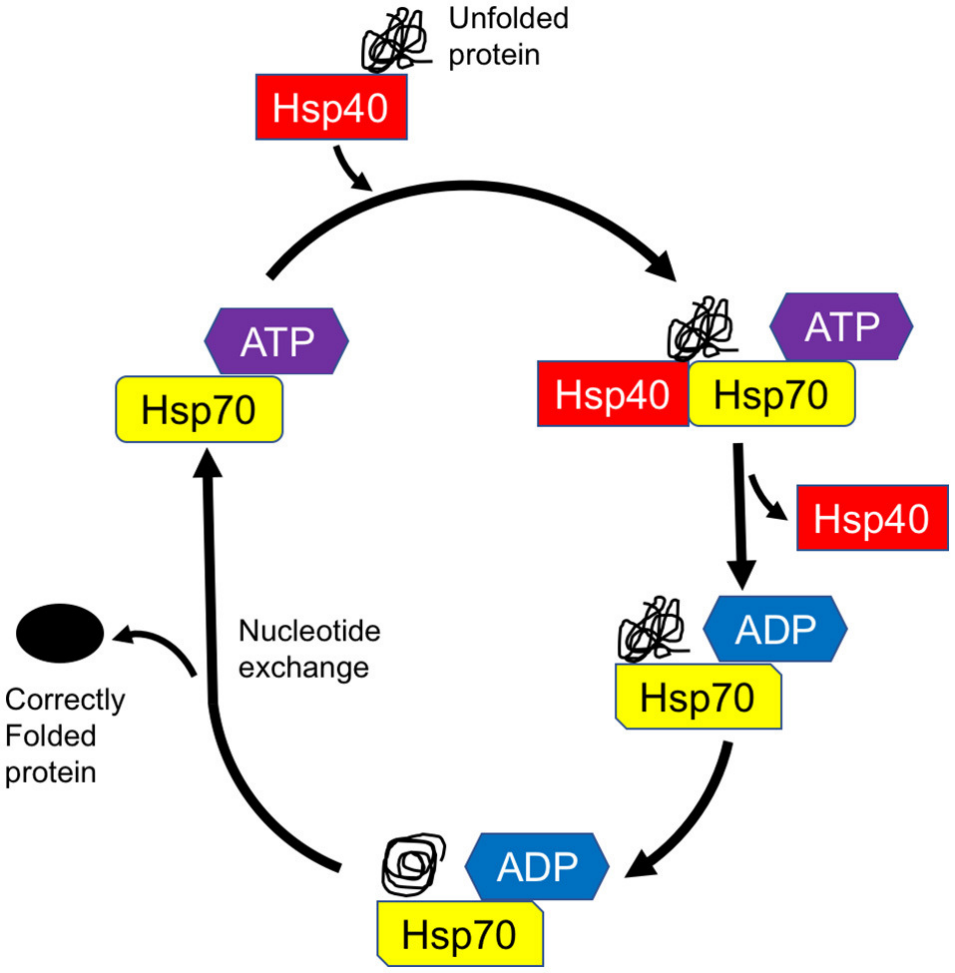
Model of chaperone-assisted protein folding by Hsp70-DnaJ/Hsp40 complex. DnaJ/Hsp40 transiently associates with unfolded protein (substrate) for delivery of the substrate to Hsp70. Through the intermediary of the J domain, Hsp40 binds to Hsp70 and promotes its ATPase activity, thus generating the ADP-bound Hsp70, which stably interacts with client protein. After nucleotide exchange, the substrate is released from Hsp70 and leaves the cycle as a correctly folded protein.

**Figure 2:**
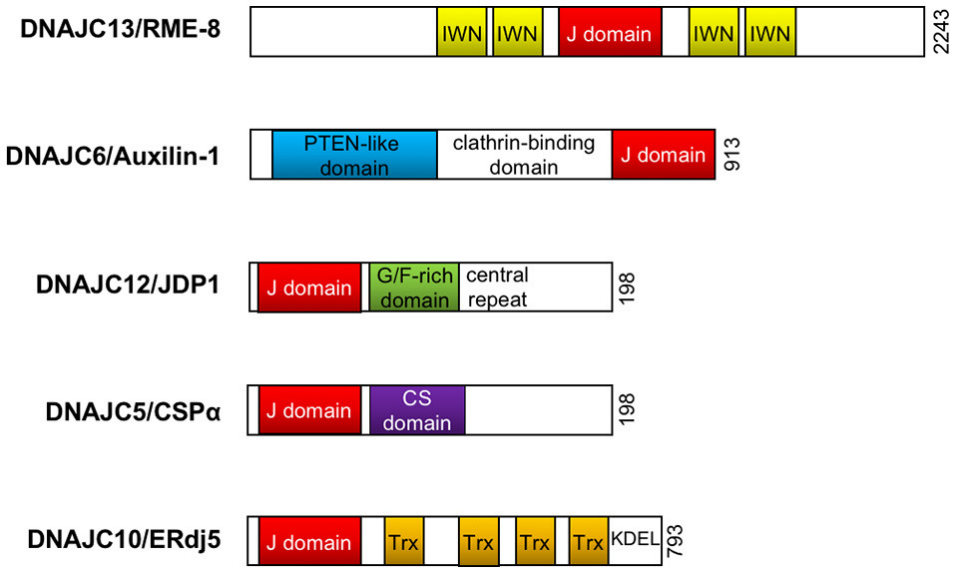
DnaJ/Hsp40 proteins implicated in Parkinson's disease. Schematic illustration of the functional domain organization of DnaJ proteins (DNAJC8/RME-8, DNAJC6/Auxilin-1, DNAJC12/JDP1, DNAJC5/CSPα, and DNAJC10/ERdj5) reviewed in this article. The illustration does not represent the actual molecular size. PTEN, phosphoinositide phosphatase; G/F, glycine/phenylalanine; CS, cysteine-string; Trx, thioredoxin; KDEL, KDEL (Lys-Asp-Glu-Leu) targeting sequence to the ER.

The complex and multifaceted functions of Hsp70 members are achieved by the assistance of their Hsp40/DnaJ co-chaperones (Qiu et al., 2006; Gorenberg and Chandra, 2017). Hsp40 family proteins were originally identified as mammalian homologs of bacterial DnaJ proteins and are subdivided into three distinct groups, i.e., DnaJA, DnaJB, and DnaJC. Structurally, DnaJ proteins consist of 4 functional domains, namely, an evolutionarily conserved J domain with a stretch of 70 amino acid residues, a glycine/phenylalanine-rich domain (G/F-rich domain), a zinc finger domain that contains CXXCXGXG motifs, and a cysteine-rich region (Qiu et al., 2006). In DnaJA and DnaJB proteins, the J domain is always located at the N-terminus which is followed by the G/F-rich domain. The DnaJC family usually lacks the G/F domain and the cysteine-rich region, and the J domain may be located at any location in the entire sequence. Through the intermediary of the J domain, Hsp40 binds to Hsp70 and promotes its ATPase activity, thus generating the ADP-bound Hsp70, which stably interacts with client proteins (Figure 1; Greene et al., 1998). Both the G/F-rich domain and c-terminal region of Hsp40/DnaJ proteins are likely to be important in their substrate recognition (Perales-Calvo et al., 2010). Although the Hsp40/DnaJ families are ubiquitously expressed, widespread distribution is also present in the central and peripheral nervous systems (Gorenberg and Chandra, 2017). Furthermore, recent genetic and biological studies suggest that Hsp40/DnaJ family genes/proteins directly or cooperatively influence on the initiation of familial Parkinson's disease (PD) and other inherited forms of parkinsonism (Figure 2 and Table 1; Gorenberg and Chandra, 2017; Hasegawa et al., 2017; Puschmann, 2017). It remains obscure how the different mutated genes could result in the progressive loss of striatal dopaminergic innervation as well as the death of nigral neurons. Nevertheless, some relationships, involving the perturbation of cellular systems, now appear to be apparent. The affected systems include synaptic transmission, endosomal trafficking, protein-quality control, and/or mitochondrial systems. In this review, we will summarize the current understandings regarding the functional roles of Hsp40/DnaJ co-chaperones in the familial forms of PD/parkinsonism and discuss how these molecules may influence on the pathological consequence of this disease.

**Table 1:** Clinicopathological features of DnaJ/Hsp40-linked PD/Parkinsonism.

**Gene**	**Transcript**	**Inheritance**	**Age at onset**	**Clinical symptoms**	**Neuroimaging findings**	**Response to L-dopa**	**LB pathology**	**Reference**
*DNAJC13*	RME-8	AD	57–76	Classic Parkinsonism, sometimes accompanied by dementia, some case clinically manifested PSP or essential tremor	Rostrocaudal striatal deficits in dopaminergic PET imaging	+	+	Appel-Cresswell et al., 2014; Vilariño-Güell et al., 2014; Lorenzo-Betancor et al., 2015; Rajput et al., 2015
*DNAJC6*	Auxilin-1	AR	7–42	Early-onset parkinsonism, occasionally accompanied by pyramidal tract sign, epilepsy, mild mental retardation and visual hallucination	Unremarkable MRI scans except for one case showing diffuse brain atrophy, abnormalities in ^18^F-DOPA-PET imaging	+/−	n.a.	Edvardson et al., 2012; Koroglu et al., 2013; Elsayed et al., 2016; Olgiati et al., 2016
*DNAJC12*	JDP1	AR	0–51	Dystonia, mental retardation, axial hypotonia, limb hypertonia, nystagmus and non-progressive parkinsonism in any combination	Unremarkable Brain MRI scans, abnormalities on ^8^F-DOPA-PET imaging was noted in one case	+	LB-negative nigral degeneration with AD pathology	Anikster et al., 2017; Straniero et al., 2017; van Spronsen et al., 2017
*DNAJC5*	CSPα	AD	26–43	Myoclonic and tonic-clonic seizure, ataxia and myoclonus, dementia with depression, premature death, sometimes accompanied by parkinsonism and pyramidal tract sign	Mild to moderate cerebro-cerebellar atrophy	n.a.	n.a.	Burneo et al., 2003; Noskova et al., 2011; Velinov et al., 2012; Cadieux-Dion et al., 2013
*DNAJC10*	ERdj5	Risk	54–70	Classic parkinsonism	n.a.	n.a.	n.a.	Yuan et al., 2016

*RME-8, receptor-mediated endocytosis-8; JDP1, J domain-containing protein; CSPα, cysteine string protein α; AD, autosomal dominant; AR, autosomal recessive; PSP, progressive supranuclear palsy; PET, positron emission tomography; MRI, magnetic resonance imaging; LB, Lewy body; n.a., not available*.

## DNAJC13/RME-8

**Figure 3:**
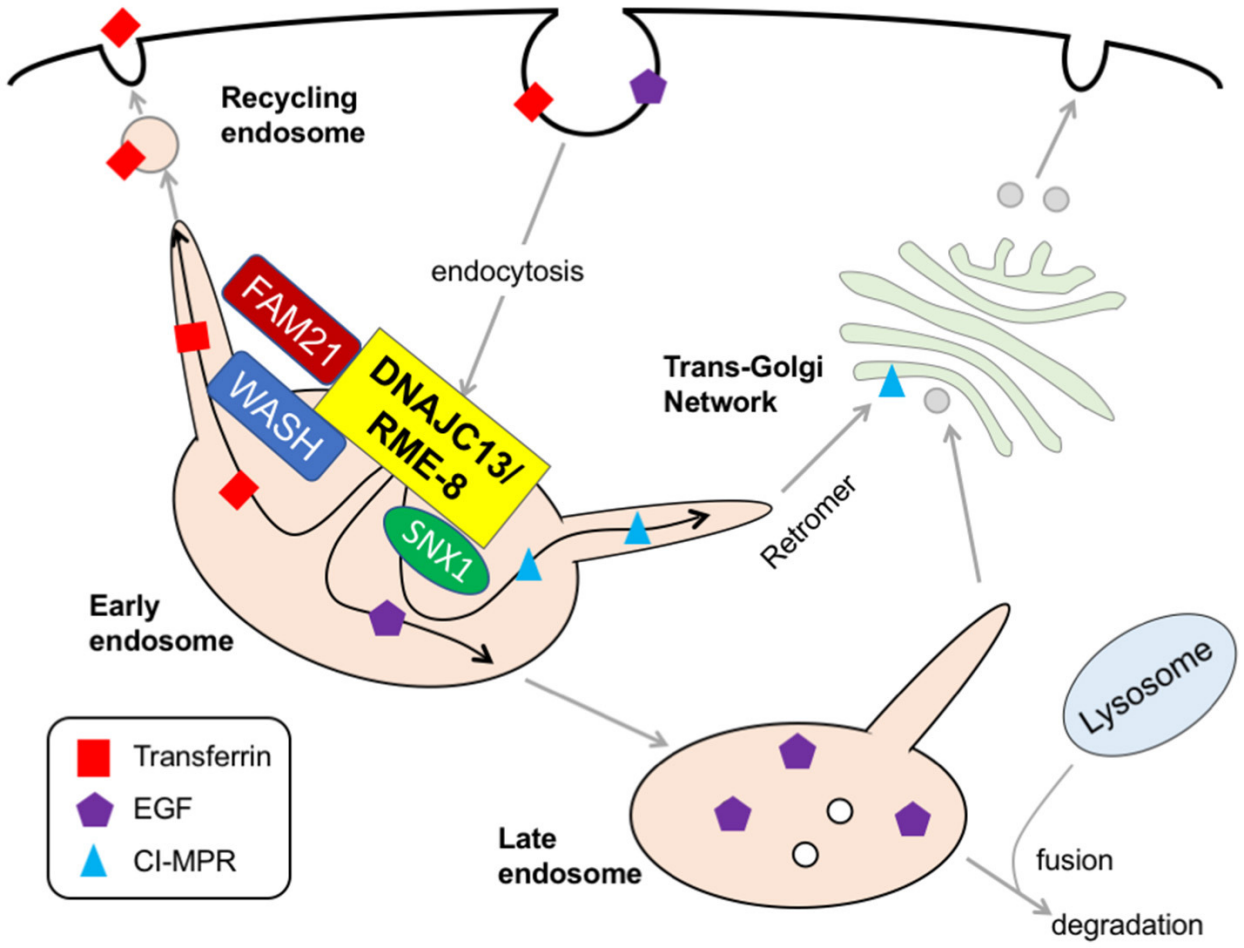
DNAJC13/RME-8. At the surface of early endosome, DNAJC13 interacts with FAM21 tail in the WASH complex and SNX1, thereby controlling the formation of tubular structure on endosome surface where a retromer-mediated cargo transport (e.g., CI-MPR) occurs. DNAJC13 also play important roles in the recycling and the degradation of transferrin and EGFR, respectively. RME-8, receptor-mediated endocytosis-8; FAM21, family with sequence similarity 21; WASH, Wiskott–Aldrich syndrome protein and Scar homolog; SNX1, sorting nexin 1; EGF, epidermal growth factor; CI-MPR, cation-independent mannose 6-phosphate receptor.

*DNAJC13* is a human homolog of *receptor-mediated endocytosis 8* (*RME-8*), which was originally identified in a genetic screening for mutants defective in the endocytic uptake of yolk protein in nematodes (Zhang et al., 2001). Recently, a p.N855S missense mutation of *DNAJC13* was reported in a large Canadian Mennonite PD pedigree with Dutch-German-Russian ancestry (Appel-Cresswell et al., 2014; Vilariño-Güell et al., 2014). The pathogenicity of the N855S mutant is supported by segregation with disease, lack of healthy controls with mutation, and the high level of sequence conservation across species; however, genetic screening in a Caucasian series consisting of 1,938 patients with clinically diagnosed PD and 838 pathologically proven Lewy Body Disease failed to detect any coding variant in exon 24 containing Asn855 in *DNAJC13* gene, indicating that mutations in this exon are not a common cause of PD or LBD among Caucasian populations (Lorenzo-Betancor et al., 2015). Subsequent genotyping in a Canadian cohort indicated that missense mutations, such as p.P336A, p.V722L, p.R1266Q, and P.T1895M, are only identified in affected members, and p.E1740Q, and p.L2170W may be associated with the risk variants for PD (Gustavsson et al., 2015). Clinically, DNAJC-linked PD (subsequently designated as PARK21) manifests as slowly progressive, late-onset, dopa-responsive typical parkinsonism with an autosomal dominant inheritance (Vilariño-Güell et al., 2014). Of note, the N855S mutation in *DNAJC13* gene has also been observed in patients with essential tremor, a monosymptomatic disorder characterized exclusively by postural- or action-type tremor, suggesting heterogeneity in the clinical manifestation with this mutation (Rajput et al., 2015). Neuropathological examination in three N855S mutation carriers indicated a brainstem or transitional type of Lewy body pathology (Vilariño-Güell et al., 2014). DNAJC13/RME-8 is a large protein with a high molecular mass of 254 kDa; it is widely expressed and is abundant in nervous tissue. Structurally, it consists of four conserved IWN repeats, which are characterized by seven invariant residues, including isoleucine, tryptophan and asparagine, and a J domain in the center region (Fujibayashi et al., 2008). An *in vitro* pull-down assay demonstrated that the J domain of RME-8 binds to Hsc70 in the presence of ADP (Chang et al., 2004). Cell biological studies have demonstrated that DNAJC13 resides in the endosomal membrane and interacts with a family with sequence similarity 21 (FAM21) tail in the Wiskott–Aldrich syndrome protein and Scar homolog (WASH) complex and sorting nexin 1 (SNX1), thereby controlling the formation of the tubular structure on the endosome surface where retromer-mediated cargo transport occurs (Freeman et al., 2014). From a functional point of view, DNAJC13 plays important roles in the cell surface recycling and lysosomal degradation of transferrin and EGFR, as well as the retrograde transport of cation-independent mannose phosphate receptor (CI-MPR; Girard et al., 2005; Popoff et al., 2009; Shi et al., 2009; Figure 3). The over-expression of p.N855S mutant, but not wild-type, in cultured cells leads to aberrant retention of transferrin in endosomes (Vilariño-Güell et al., 2014), which indicates that a PD-linked DNAJC13 mutation may confer a toxic gain-of-function and hampers endosomal cargo trafficking. Intriguingly, immunofluorescence and co-immunoprecipitation studies demonstrate that vacuolar protein sorting 35 (VPS35), a vital element of retromer that is established as a causal gene for PARK17 PD, is co-localized with DNAJC13 in a primary cortical neuron culture obtained from mice (Vilariño-Güell et al., 2014). Further studies using appropriate animal models are required to better understand the precise molecular mechanisms by which *DNAJC13* mutation leads to the dopaminergic degeneration with Lewy body pathology. In an independent study, Deng et al. identified loss-of-function mutations in *TMEM230* gene in the same Canadian Mennonite group that Vilariño-Güell and his colleagues previously identified mutations in DNAJC13 (Deng et al., 2016). The *TMEM230* transcript localizes to the secretory and recycling vesicle in the neuron and may be involved in synaptic vesicle trafficking and recycling. Another study has shown that TMEM230 is required for Rab8a-mediated transport of secretory vesicles and retromer-mediated cargo trafficking (Kim et al., 2017). Further discussion and reassessment are needed to understand this complex situation.

## DNAJC6/Auxilin-1

**Figure 4:**
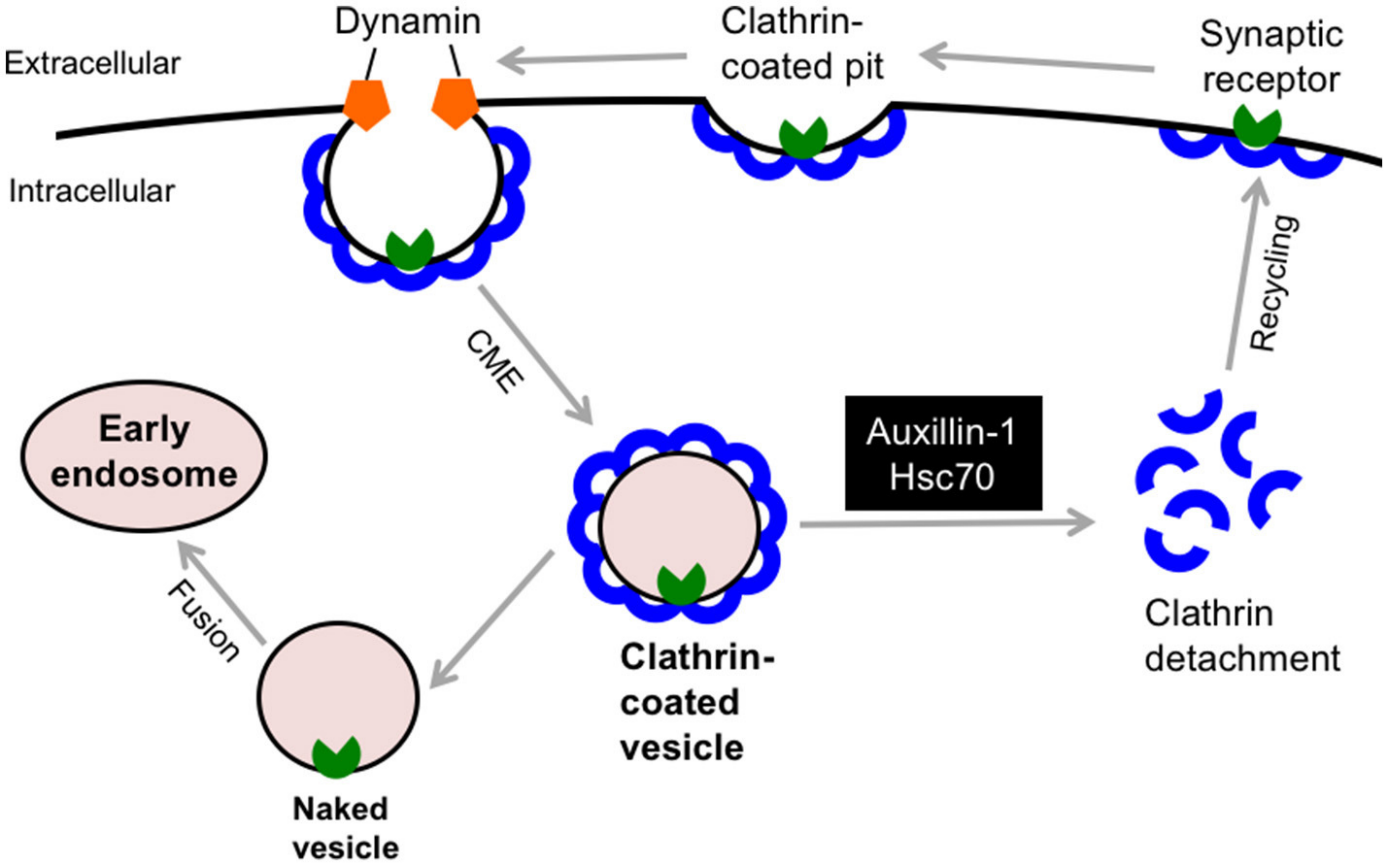
DNAJC6/Auxilin-1 regulates the clathrin-mediated endocytosis. DNAJC6/Auxilin-1 is highly expressed in nerve terminals and plays a crucial role in clathrin uncoating during synaptic receptor endocytosis. CME, clathrin-mediated endocytosis; Hsc70, heat shock cognate 70.

In 2012, deleterious (c.801-2 A>G) and truncating (p.Q734X) mutations in the *DNAJC6* gene were discovered in two consanguineous families with Palestinian and Turkish origins, respectively (Edvardson et al., 2012; Koroglu et al., 2013). The clinical phenotypes of DNAJC6-linked familial parkinsonism (termed PARK19) are characterized by a juvenile-onset, progressive parkinsonism, which includes bradykinesia, muscle rigidity, resting tremor, hypomimia, and postural instability. In addition to extrapyramidal signs, four members of the Turkish family exhibited hypomentia, pyramidal tract signs and/or seizures (Koroglu et al., 2013). Patients in the Palestinian family had dopa-refractory parkinsonism, whereas affected members in the Turkish family showed a favorable response to levodopa replenishment therapy. Subsequent genetic studies identified 2 different homozygous mutations in the *DNAJC6* gene, i.e., a missense mutation (p.R927G) and a putative splice site mutation (c.2223A-T; Olgiati et al., 2016). These families accounted for 2 (2.2%) of 92 probands (mean age at onset: 54.65; range 19–84) with autosomal recessive PD who underwent *DNAJC6* genetic screening. These patients showed milder phenotype compared to the patients with truncating mutations probably due to some residual activity of DNAJC6. Another novel non-sense mutation (exon 16, p.Q789^*^) has also been reported in a consanguineous family with juvenile-onset PD (Elsayed et al., 2016). Of interest, a patient in this family manifested rigid-akinetic form of parkinsonism with visual hallucinations, which are frequently observed non-motor symptom in sporadic PD and dementia with Lewy bodies (Shoji et al., 2014). Because there is variation in the responsiveness of dopamine-replacement therapy, the term “parkinsonism” rather than PD would be suitable in *DNAJC6*-linked movement disorder. The *DNAJC6* encodes the neuron-specific isoform of the co-chaperone auxilin-1, which has a crucial role in the detachment of the clathrin-coat after clathrin-mediated endocytosis (CME, Figure 4). Auxilin-1 is a 100 kDa protein and structurally consists of 3 distinct domains: the N-terminal phosphoinositide phosphatase (PTEN) like domain (residue 40–421), which is required for the recruitment to a clathrin-coated pit, the central clathrin-binding domain, and a C-terminal J domain, which enables its interaction with Hsc70 (Lee et al., 2006). The c.801-2 A>G homozygous splice-site mutation in *DNAJC6* yields two misspliced abnormal transcripts that lack either a significant part of the J domain or the PTEN-like domain, which indicates that this pathological mutation results in the defect of functionally active auxilin-1 (Edvardson et al., 2012). The homozygous mutation p.Q734X deletes the 180 amino acid residues of Auxilin-1 at the C-terminus, which are considered important for supporting Hsc70 function in clathrin dissociation from coated vesicles (Koroglu et al., 2013). Moreover, an *in silico* structural analysis predicted that that the p.R927G missense mutation in the J domain reduces the positive charge on the protein surface, which would be involved in the molecular interaction of auxilin-1 with Hsc70 or other partners (Olgiati et al., 2016). Interestingly, common variants in *cyclin-G-associated kinase* (*GAK*), which encodes auxilin-2/DNAJC26, a ubiquitously expressed paralog of auxilin, are also considered a risk factor for idiopathic PD (Pankratz et al., 2009). Auxilin-null mice exhibited a high mortality rate in early life; however, they did not exhibit no obvious neuropathological alterations in the midbrain (Yim et al., 2010). These mice showed higher GAK levels in the brain; however, this compensation appeared to be insufficient for the lack of auxilin in clathrin uncoating since they exhibited an aberrant retention of clathrin-coated vesicles and empty clathrin cages at the synaptic terminals, which may be attributed to the parkinsonian phenotypes. Further investigation using genetically modified animal models is warranted to better understand the role of DNAJC6 in synaptic dopamine function.

## DNAJC12/JDP1

DNAJC12, a member of the Hsp70/Hsc70 co-chaperone proteins, has the J domain and a highly conserved C terminus; however, it lacks other canonical domains present in DnaJ family proteins. This protein is a mammalian ortholog of the J domain-containing protein (JDP) originally identified in *Drosophila* and is thus termed JDP1 (Hahn et al., 1999). In mammals, two transcript variants of DNAJC12/JDP1 have been identified: the primary isoform is 1.2 kb and encodes 198 amino acids, and the other isoform is 0.7 kb and is composed of 107 amino acids. Northern blot analysis showed that the major transcript of mouse JDP1 is ubiquitously expressed in various organs, including the brain (Hahn et al., 1999). Recently, biallelic mutations [del. 6943 (c.298-968_503-2603del) and p.R72P] of the *DNAJC12* gene have been identified as causes for phenylketonuria (PKU), the most frequent inherited metabolic disorder (Anikster et al., 2017). Segregation analysis confirmed autosomal-recessive inheritance in all families. Furthermore, a subsequent study of the genotype-phenotype correlation in *DNAJC12* mutations demonstrated that several homozygous null variants (c.187A>T and c.79-2A>G) in *DNAJC12* present young-onset, dopa-responsive non-progressive parkinsonism as the cardinal symptom and mild cognitive decline without apparent dystonia (Straniero et al., 2017). The autopsy studies of the proband's brain showed depigmentation in the substantia nigra, and no α-synuclein-positive Lewy pathology was observed. Thus, the PKU with the *DNAJC12* mutation should be considered as a differential diagnosis of early-onset parkinsonism, since a strict diet therapy with low phenylalanine may prevent the progression of the disease. Most patients with PKU have mutations in a gene that encodes phenylalanine hydroxylase (PAH), a rate-limiting enzyme of the metabolic pathway that degrades excess phenylalanine to tyrosine (Blau, 2016). Patients with PKU do not show any abnormalities at birth; however, if left untreated, the accumulation of phenylalanine and the insufficient level of tyrosine insidiously lead to psychomotor retardation, microcephaly, epilepsy, generalized dystonia, and hypopigmentation in the skin and hair (Al Hafid and Christodoulou, 2015). A limited number (up to 2%) of PKU cases are caused by a deficiency of tetrahydrobiopterin (BH_4_), a co-factor of PAH and other members of aromatic amino acid hydroxylases (AAAHs), such as tyrosine hydroxylase (TH) and tryptophan hydroxylase (TPH), which are required for the biosynthesis of dopamine and serotonin, respectively (Blau, 2016). Affinity capture-mass spectrometry (AC-MS) and co-immunoprecipitation demonstrated that DNAJC12 interacts with AAAHs including PAH, TH, and peripheral and neuronal isoforms of TPH (Huttlin et al., 2015; Anikster et al., 2017). A molecular interaction between PAH and JDP, the ortholog of DNAJC12 in fruit fly, was also detected in the two-hybrid-based protein-interaction datasets of *Drosophila* (the Biological General Repository for Interaction Datasets, BioGRID; Giot et al., 2003). Furthermore, analysis of cerebrospinal fluid from affected individuals showed deficiencies of several catecholamines and their metabolites, including dopamine, serotonin, HVA, and 5-HIAA (van Spronsen et al., 2017). These findings suggest the importance of DNAJC12 for proper function of enzymes involved in catecholamine biosynthesis. The c.158-2A>T splice variant and entire deletion in exon 4 (del. 6943) are considered to be null mutants as the DNAJC12 protein was below the detection level in the fibroblasts obtained from individuals carrying these mutations (Anikster et al., 2017). Moreover, the p.R72P missense variant located in the J domain appears to affect the stability of DNAJC12 as the Arg72 residue is well-conserved across species and is indispensable for maintaining the 3D structure of the J domain through the interactions with the Ser25 residue (Anikster et al., 2017).

## DNAJC5/CSPα

DNAJC5, also referred to as cysteine string protein α (CSPα), is a 34 kDa protein and synaptic co-chaperone of the DnaJ/Hsp40 family (Burgoyne and Morgan, 2015). The name originates from the existence of a cysteine string domain that consists of 13–15 heavily palmitoylated cysteine residues within 25 amino acids. Structurally, DNAJC5/CSPα is divided into the following domains: a phosphorylation site for protein kinase A at an N-terminus, a conserved J domain, an adjacent linker region, the cysteine string domain, and a less conserved C-terminal domain (Burgoyne and Morgan, 2015). Mutations (p.L116del and p.L115R) in the cysteine string domain of *DNAJC5* have previously been identified as causes for an autosomal-dominant, adult-onset neuronal ceroid lipofuscinosis (ANCL, also referred to as Kufs disease), a neurodegenerative disorder characterized by abnormal accumulation of fluorogenic lipids, granular substances not only in the neuronal cells in the brain but also in some other tissues (Noskova et al., 2011; Velinov et al., 2012; Cadieux-Dion et al., 2013). Sanger sequencing revealed that p.L116del mutation was the only indel completely co-segregating with the phenotype in the pedigree. The clinical picture of individuals who carry pathogenic *DNAJC5* mutations consists of tonic-clonic and myoclonic epileptic seizure, ataxia, and dementia with premature death. In some cases, patients subsequently began to manifest parkinsonism (Burneo et al., 2003; Cadieux-Dion et al., 2013). The over-expression of EGFP-tagged DNAJC5 in the subclone of murine Cath.a differentiated neuronal cell line (CAD5) demonstrated cell surface localization of wild-type DNAJC5, whereas the p.L116del and p.L115R mutant proteins showed diffuse cytoplasmic localization in addition to aberrant accumulation in the ER and Golgi apparatus (Noskova et al., 2011). As expected, these mutants were less palmitoylated than wild-type DNAJC5, which may lead to impaired membrane tethering of DNAJC5/CSPα and a reduced protein level of DNAJC5/CSPα in the brains of affected individuals. Furthermore, p.L116del and p.L115R mutations in the cysteine-string domain cause DNAJC5/CSPα to form high molecular weight SDS-resistant aggregates, which are also present in post-mortem brain tissue from patients, suggesting a cluster of palmitoylated cysteines are essential for aggregation of CSPα (Diez-Ardanuy et al., 2017). In agreement with this, transgenic zebrafish model expressing the human mutant *DNAJC5* gene under the control of a zebrafish neuron-specific promoter demonstrated mutant DNAJC5 protein aggregates in the affected neurons (Yao et al., 2017). However, the precise molecular mechanisms by which the mutant DNAJC5 leads to neurodegeneration accompanied by abnormal accumulation of autofluorescence materials in neuronal tissues still remain obscure. Accumulating evidence suggests that the DNAJC5/CSPα behaves as a critical regulator of synaptic proteostasis. Together with a small glutamine-rich tetratricopeptide repeat domain protein (SGT), DNAJC5/CSPα forms an enzymatically active chaperone complex and binds misfolded client proteins on synaptic vesicles, thereby preventing the buildup of misfolded proteins in the nerve terminal (Donnelier and Braun, 2014). For example, the DNAJC5-Hsc70-SGT chaperone complex stabilizes synaptosomal-associated protein 25 (SNAP-25) and facilitates its ability to assemble into the soluble NSF attachment protein receptor (SNARE) complex (Sharma et al., 2011). Intriguingly, the over-expression of α-synuclein, a culprit protein in PD, protects DNAJC5/CSPα-deficient mice from neurodegeneration (Chandra et al., 2005), which suggests that α-synuclein may compensate for the loss of DNAJC5/CSPα in the nervous system. This finding has been corroborated by a recent study showing that DNAJC5/CSPα removes neurodegeneration-related toxic proteins (e.g., abnormally expanded huntingtin and mutant superoxide dismutase 1) from neurons via extracellular vesicles (Deng et al., 2017).

## DNAJC10/ERdj5

A recent comprehensive analysis of genetic variants in a well-characterized Han-Chinese cohort with sporadic PD demonstrated that the *DNAJC10* gene variant rs13414223 decreased the risk of PD (Yuan et al., 2016). The statistical differences in genotypic and allelic frequencies between the PD and the controls were *p* = 0.004 and 0.002 (odds ratio = 0.652), respectively. The gene of *DNAJC10* encodes the 90 kDa ER-resident co-chaperone ERdj5, which is a component of ER-associated degradation (ERAD), a quality-control machinery by which unfolded/misfolded proteins are degraded in eukaryotic cells (Ushioda et al., 2008). The ERdj family comprises five members, and each member contains a conserved N-terminal J domain; ERdj5 is the only member that has thioredoxin (Trx)-like domains with catalytic active CXXC motifs (Cunnea et al., 2003). A KDEL tetrapeptide sequence is present at the C terminus of DNAJC10/ERdj5, possibly mediating the targeting to the ER (Stornaiuolo et al., 2003). Via its reductase activity, DNAJC10/ERdj5 cleaves the disulfide bonds of misfolded proteins and thereby accelerates ERAD through the associations with EDEM (ER degradation-enhancing alpha-mannosidase-like protein) and the ER chaperone BiP/GRP78 (Ushioda et al., 2008). DNAJC10/ERdj5 is ubiquitously expressed across brain areas, including the cerebral cortex, striatum, hippocampus, hypothalamus, cerebellar cortex, and brainstem (Cunnea et al., 2003), which are the anatomical regions of neuronal cell loss and Lewy body formation in PD (Braak and Braak, 2000). Intriguingly, *C. elegans* DJ-27, an ortholog of mammalian ERdj5, showed a protective effect against aggregate formation, behavior abnormalities, and mitochondrial fragmentation in worm models of human Alzheimer, Parkinson, and Huntington diseases (Munoz-Lobato et al., 2014); these findings suggest that DNAJC5 may counteract protein misfolding/aggregation toxicity in neuronal cells. Studies in higher model organisms, such as vertebrates, would be useful and advantageous to figure out how the DNAJC10/ERdj5 mutation can be involved in the pathogenesis of PD.

## Concluding remarks

Although more than 90% of PD cases occur sporadically, both familial and sporadic PD share common pathological features, such as cytoplasmic inclusions and dopaminergic cell loss (Hasegawa et al., 2004; Takeda et al., 2006). Evidence from recent genetic studies in rare familial forms of PD indicates that DnaJ/Hsp40 molecular chaperones are profoundly involved in the pathogenesis of PD. The diverse functions of the DnaJ/Hsp40 family in protein folding/unfolding, membrane trafficking, synaptic modulation and mitochondrial function are considered to not only affect the dopaminergic neurotransmission but also concomitantly influence on the PD-related neuropathological changes, such as nigral cell loss and Lewy body formation. HSPs are known to be localized with noxious proteinaceous aggregates in various neurodegenerative diseases including PD, Alzheimer's disease and prion disease (Matsuzaki et al., 2004; San Gil et al., 2017). Interestingly, several studies have shown that selective upregulation of HSPs such as DnaJ/Hsp40 and Hsp70 prevents clinicopathological progression in a variety of cellular and animal models (Popiel et al., 2012; Gao et al., 2015; Takeuchi et al., 2015). Thus, genetic as well as pharmacological manipulation of specific DnaJ/Hsp40 function may provide beneficial effects on protein and cellular homeostasis in neurodegenerative conditions. Deciphering the precise modes of DnaJ/Hsp40 functions in the pathological cascades in PD will shed light on the pathogenic mechanisms involved and may provide important clues regarding the disease-modifying strategies for this devastating neurodegenerative disease.

## Author contributions

All authors (TH, SY, NS, JK, and MA) participated in the discussion of the paper. TH and SY mainly wrote the manuscript.

### Conflict of interest statement

The authors declare that the research was conducted in the absence of any commercial or financial relationships that could be construed as a potential conflict of interest.
